# Hypopigmented Mycosis Fungoides in Type V Skin: A Report of 5 Cases

**DOI:** 10.1155/2011/190572

**Published:** 2011-12-20

**Authors:** Ranthilaka R. Ranawaka, Priyanka H. Abeygunasekara, M. V. Chandu de Silva

**Affiliations:** ^1^Department of Dermatology, Teaching Hospital Anuradhapura, Anuradhapura, Sri Lanka; ^2^Department of Pathology, Teaching Hospital Anuradhapura, Anuradhapura, Sri Lanka; ^3^Department of Pathology, Faculty of Medicine, University of Colombo, Kinsy Road, Colombo 8, Sri Lanka

## Abstract

Five patients with type V skin were studied to describe the clinical manifestations, pathological features, and treatment response in hypopigmented mycosis fungoides (HMF). The mean age of patients was 22.4 years at diagnosis, with a mean of 36 months of diagnostic delay. Two were children aged 11 and 13 years. Skin patches were limited to sunlight-covered body areas. In tropical climate, exposure to natural sunlight possibly cured the lesions on sun-exposed areas at early stage of onset. HMF may frequently be misinterpreted as eczema, vitiligo, or progressive macular hypomelanosis clinically and histopathologically as seen in our case series.

## 1. Introduction

Mycosis fungoides (MF), the most common form of cutaneous T-cell lymphoma, is predominantly a disease of older patients. Approximately 75% of patients are diagnosed after age 50 years [1].

Hypopigmented mycosis fungoides (HMF) is an atypical, rare and unique variant of mycosis fungoides characterized by solely hypopigmented patches or in combination with erythematous patches or plaques. Its course is indolent for several years and thus source of delayed diagnosis [1–3]. The disease has predilection for dark-skinned individuals of African, West Indian, or Asian origin. The incidence of HMF in white Caucasian is rare with few cases reported [4, 5]. Patients with HMF are younger (mean of 29 years) than classical MF (mean of 63 years) [3–8]. Overall survival rate and disease-specific survival rates at 5 and 10 years were 100% in hypopigmented MF and stage 1A disease, and disease progression at 5 and 10 years was 0% in HMF in a study published by Wain et al. [7].

Topical steroids [9] and psoralen plus ultraviolet A (PUVA) [10, 11] were the treatment of choice in the majority of reported cases. Narrowband UVB (NB-UVB) phototherapy had been introduced recently as a successful treatment option for early-stage disease (stage IA, IB and IIA) [11–15].

This case series was retrospectively studied to describe the clinical manifestations, pathological features, and treatment response in 5 patients with hypopigmented MF who presented to our department over 3-year period.

## 2. Patients and Methods

 The diagnosis was established by the appearance of skin lesions and confirmed histopathologically. At least, two biopsies were obtained from each case from clinically suspicious areas. The skin biopsies were routinely processed; 4-5 micron thick sections were stained with haematoxylin-eosin stain and examined under light microscopy. All the skin biopsies were independently reviewed by two pathologists. Staging was done according to TNM classification of mycosis fungoides.

Patients were treated with PUVA (*n* = 1), narrow band-UVB (*n* = 3), and topical steroids (*n* = 1). The response to therapy was assessed as follows: complete clinical response (CR), when complete disappearance of lesions for at least 1 month; partial clinical response (PR), when greater than 50% improvement of skin lesions; no response (NR), when unchanged of skin lesions.

## 3. Case Series

2 men and 3 women were studied. All were Fitzpatrick skin type V. The mean age of patients' was 22.4 years at diagnosis, with a mean of 36 months duration of illness before presentation. Two were children aged 11 and 13 years—Table 1.

### 3.1. Clinical Presentations

#### 3.1.1. Patient 1: Figures 1(a) and 1(b)

A 13-year-old girl had skin patches over buttocks and upper thigh involving bilaterally for 3 years duration. They were asymptomatic, hypopigmented, oval or irregular, well-defined, size ranged from 1–6 cm diameters. There were vitiligo-like lesions on buttocks.

#### 3.1.2. Patient 2: Figures 2(a) and 2(b)

A 29-year-old man had finely wrinkle, slightly scaly skin patches on trunk, upper and lower limbs for 3 years. They were not indurated, and there were no telangiectasia and no sensory impairment. Size ranged from 6 to 8 cm diameters.

#### 3.1.3. Patient 3: Figure 3

An 11-year-old boy had localized depigmented patches of 5–8 cm diameter, on buttocks and posterior upper thigh, which were well demarcated simulating vitiligo. He was misdiagnosed clinically and histopathologically as eczema and vitiligo for 3 years. His skin lesions were atrophic at the time of examination possibly due to long-term application of potent topical steroids.

#### 3.1.4. Patient 4: Figures 4(a) and 4(b)

A 30-year-old woman had hypopigmented discrete patches of 1-2 cm diameter on flexure aspect of all four limbs, trunk and breasts for 3 years. She was repeatedly treated for pityriasis versicolor (PV) and progressive macular hypomelanosis (PMH) without success.

#### 3.1.5. Patient 5: Figure 5

A 29-year-old woman presented with discrete hypopigmented patches of 4–6 cm diameter on limbs and trunk. These were treated as PV and vitiligo by many physicians over 5 years.

All, except patient 2, had skin lesions limited to sunlight-covered body areas. None of them had hepatosplenomegaly at the time of diagnosis. General examination was unremarkable in all.

### 3.2. Histopathology

The biopsy specimens of skin lesions showed atypical lymphocytes with hyperchromatic nuclei in the upper dermis with epidermotropism and formation of Pautrier's microabscesses. Three cases (patients 1, 3, and 5) showed extensive changes with marked epidermotrophism of atypical lymphocytes and formation of numerous (>3) Pautrier's microabscesses (Figures 6(a) and 6(b)). The epidermal changes in other two were not so extensive with formation of at least one microabscess (Figures 7 and 8). The dermis contained a dense band-like upper dermal infiltrate in patient 3 with atypical lymphocytes. Others showed patchy atypical lymphocytic infiltrate in dermis. 

Hemogram, routine blood chemistry, ultrasonography of abdomen and pelvis, and chest X-ray were normal. Blood pictures did not show atypical lymphocytes. Two patients (patients 2 and 5) who underwent bone marrow aspiration and trephine biopsy did not reveal significant pathology. Others were not assessed with bone marrow investigations.

### 3.3. Staging

Patient 3 was in stage 1A (skin patches or plaques with <10% skin surface involvement), others were in stage 1B (skin patches or plaques with >10% skin surface involvement) at the time of diagnosis. Although there were palpable axillary lymph nodes of 0.5–1 cm diameter in patient 1, they were not accessible surgically and were not categorized to stage IIA (skin patches or plaques with palpable lymph nodes).

### 3.4. Treatment Response

**Figure 2:**
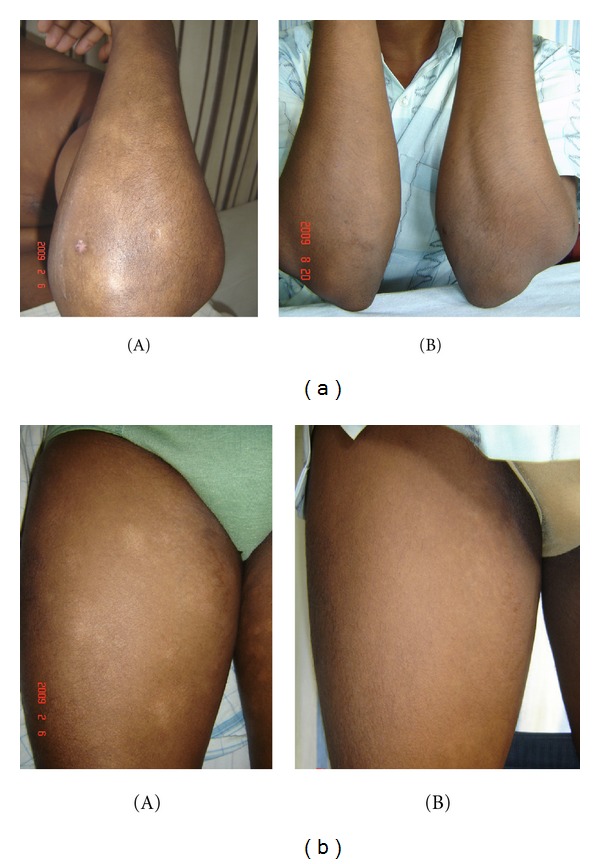
(a) A 29-year-old man had hypopigmented, finely wrinkle, slightly scaly patches without telangiectasia or induration on bilateral forearm and trunk. (b) Similar lesions on thigh bilaterally. (A) Before therapy and (B) complete clinical response after 30 sessions of PUVA (patient 2).

**Figure 4:**
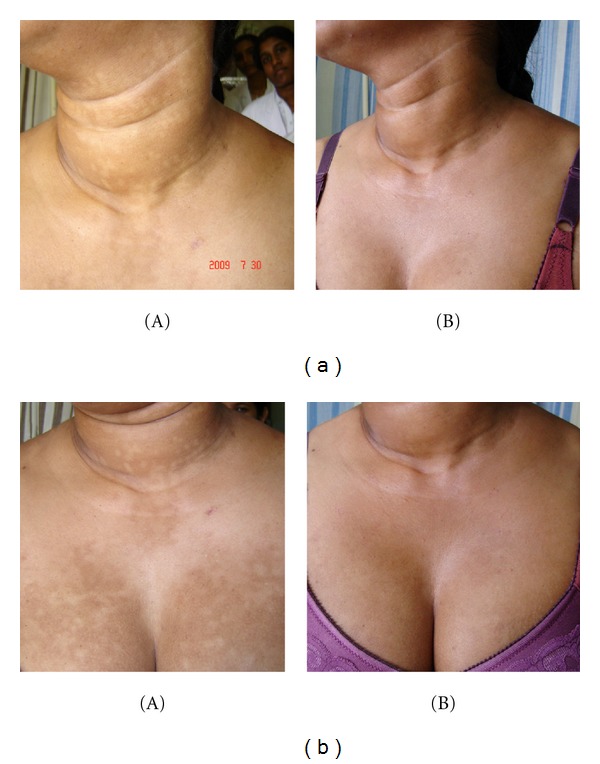
(a) A 30-year-old woman had hypopigmented patches on sunlight covered body sites. Note these patches extend to neck and (b) breast which is less likely in progressive macular hypomelanosis. (A) before therapy (B) showing almost complete clinical response after 40 sessions of narrow-band UVB. Note some hypopigmented lesions persist under the chin and neck (patient 4).

**Figure 5:**
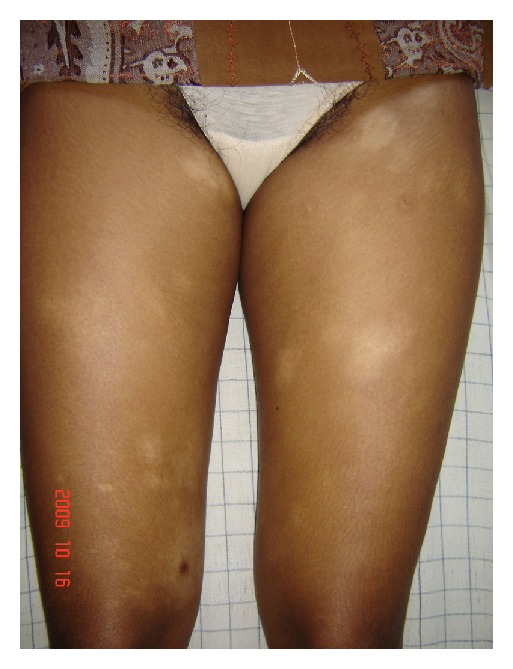
A 29-year-old woman showing hypopigmented skin patches on thigh after 32 sessions of narrow band-UVB therapy (patient 5).

**Figure 6:**
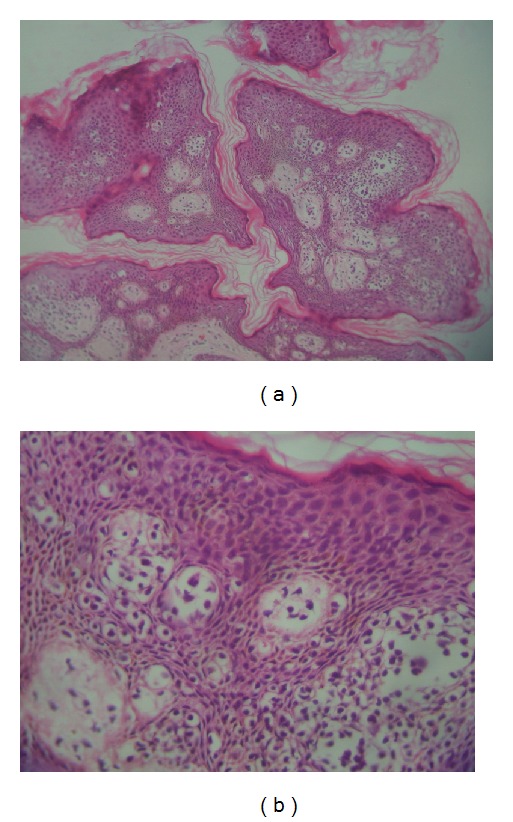
(a) The skin biopsy from buttocks (patient 3) shows extensive epidermal changes with marked epidermotrophism of lymphocytes (Hematoxylin-eosin stain, original magnification ×100). (b) Greater magnification highlights atypical lymphocytes and formation of numerous (>3) Pautrier's microabscesses (H&E stain, 400x).

**Figure 7:**
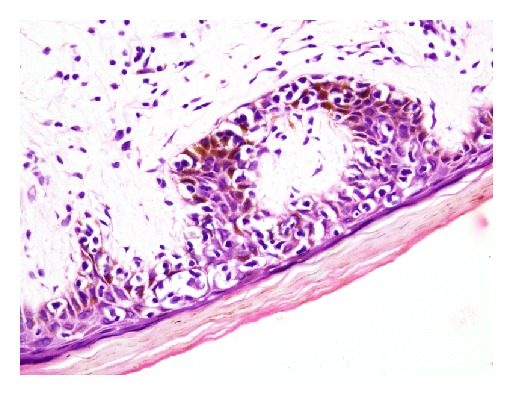
Atypical lymphocytes showing epidermotropism and formation of Pautrier's microabscesses (H&E stain, 400x) (patient 2).

**Figure 8:**
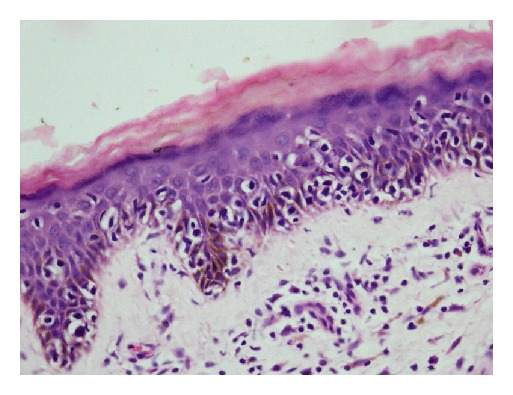
Aypical lymphocytes in lacunae in the epidermis (H&E stain, 400x) (patient 4).

PUVA showed CR in patient 2 (Figures 2(a) and 2(b)). Narrow-band UVB showed CR in patients 1 and 4 (Figures 4(a) and 4(b)), and PR in patient 5 (Figure 5) within 30–48 sessions. None of them develop any systemic or local side effects during phototherapy. Patient 3 was managed with topical steroids and showed partial clinical response within 12 months of followup.

## 4. Discussion

Most patients of HMF are misdiagnosed as having other hypopigmented skin disorders such as vitiligo, pityriasis alba, pityriasis versicolor, postinflammatory hypopigmentation, pityriasis lichenoides chronica, small plaque parapsoriasis, progressive macular hypomelanosis, and multibacillary leprosy as in our case series [16–18]. Progressive macular hypomelanosis (PMH), which is a frequently found benign skin condition in Asians, usually start on the back of the trunk in and around midline, rarely extending to buttocks, proximal limbs, and neck. PMH is characterized histologically by diminished pigment in the epidermis and a normal-looking dermis [19].

Since Sri Lanka is a tropical country with 10–12 hours sunlight, exposure to natural sunlight possibly has cured the lesions on sun-exposed areas at early stage of onset as seen in our four patients.

Asymptomatic hypopigmented skin patches uniformly distributed in neck, limbs, trunk, breasts, and buttock alarmed consultant dermatologists in our case series. Some lesions showing atrophic, finely wrinkle, slightly scaly surface (patient 2) added to the clinical acumen.

In early-stage MF clinical diagnosis supersede histopathological confirmation by many years. These patients underwent several skin biopsies before histopathological confirmation. Histopathology of early-stage MF may be debatable where two pathologists would contradict. Clinicopathologic correlation is ultimately essential to make accurate diagnosis of early MF and its histologic mimickers [16].

In contrast to conventional MF, in which the neoplastic cells are CD4+ in the vast majority of cases, the neoplastic cells in hypopigmented MF have a CD8+ T-cell phenotype [7, 20, 21]. We did not study immunophenotype of the infiltrating cells, as the facility was not available in Sri Lanka.

The pathogenesis of hypopigmentation in HMF, though still unclear, probably due to cytotoxic effect of melanosomal-antigen-specific CD8+ neoplastic T lymphocytes resulting in dysfunction and/or loss of melanocytes in the epidermis [20].

Since early-stage MF (stage 1A, 1B, IIA) has generally a good prognosis, and long-term survival rates with current therapies (UVB, PUVA, topical nitrogen mustard, electron beam radiotherapy) are similar, there is concern regarding their potential side effects. It has been reported that the same effective UVB dose is safer than PUVA in terms of carcinogenicity, and that it produces fewer side effects [11]. Narrowband (311-nm) UVB therapy was found to be more effective than broadband UVB therapy in small plaque parapsoriasis and early-stage MF [12–15]. Though HMF has an indolent course for many years, recurrences are reported; therefore, patients will be followedup for life.

In conclusion, from the 5 patients reported to date, it may be said that, HMF is characterized by early onset, occurrence in dark-skinned individuals, and good response to phototherapy. In tropical climate, exposure to natural sunlight may possibly have cured the lesions on sun-exposed areas at early stage of onset. Since it may mimic several other hypopigmented skin disorders clinically and histopathologically, clinicopathologic correlation is ultimately essential to make accurate diagnosis.

## Figures and Tables

**Figure 1:**
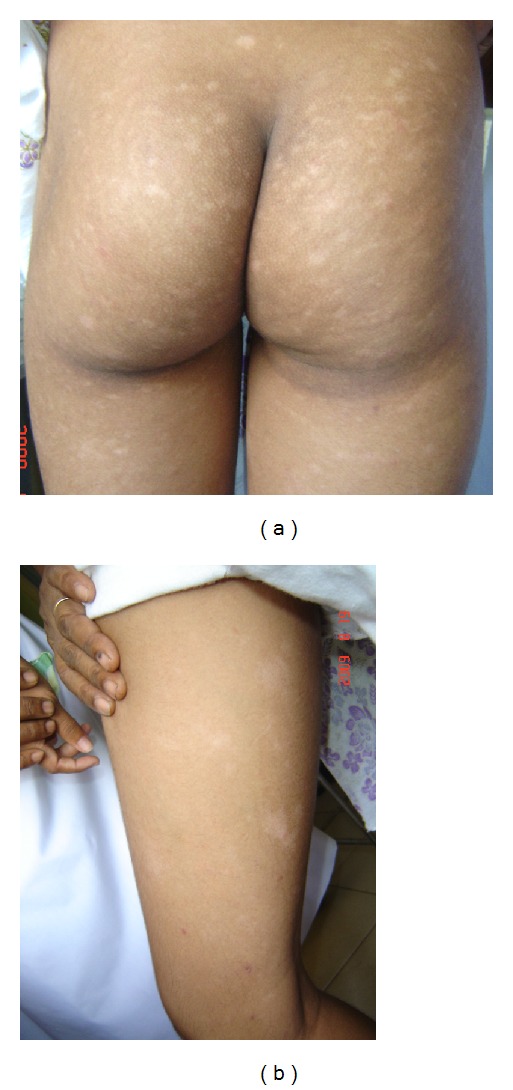
A 13-year-old girl presented with asymptomatic, hypopigmented, and a few vitiligo-like macules on buttocks and upper thigh involving bilaterally, which had been there for 3 years duration (patient 1).

**Figure 3:**
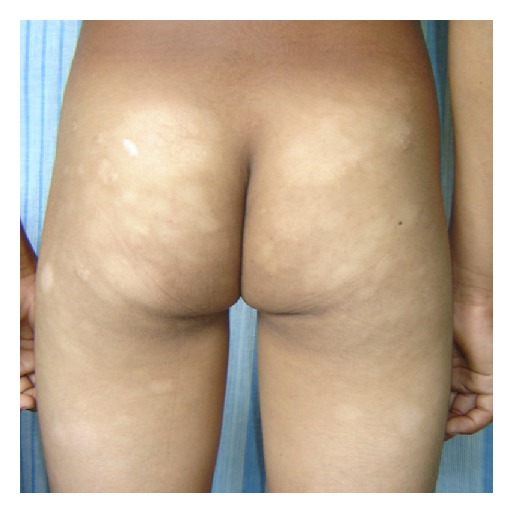
An 11-year-old boy had localized depigmented patches on buttocks and posterior upper thigh simulating vitiligo. He was misdiagnosed clinically and histopathologically as eczema and vitiligo for 3 years. This photograph was taken 8 months after potent topical steroid therapy (patient 3).

**Table 1:** Hypopigmented mycosis fungoides: patient demographics, clinical manifestations, and response to therapy.

Patients' index number	Age at diagnosis/onset (years)	Gender	Initial diagnosis	Site of skin involvement	% of skin involvement at the time of diagnosis	lymph nodes (LN), liver (Liv), spleen (Sp)	Stage at diagnosis	Previous treatments	Type of therapy given	Response to therapy
1 (Figure 1)	13/10	F	PV	Both lower and upper limbs, vitiligo-like lesions on buttocks	30%	L/axillary LN 0.5–1 cm diameter	1B	Topical antifungal	NB-UVB 32 (3x/wk)	CR

2 (Figure 2)	29/26	M	PV	All 4 limbs, buttock, a few patches on trunk	40%	None	1B	Topical antifungal	PUVA 36 (2x/wk)	CR

3 (Figure 3)	11/08	M	Eczema, vitiligo	Vitiligo-like patches on buttocks	<10%	None	1A	Topical steroids, 10% coal tar lotion and sun light exposure	Potent topical steroids daily for 12 months	PR

4 (Figure 4)	30/27	F	PV, PMH	All 4 limbs flexure aspect, anterior and back of trunk, breast	30%	None	1B	Topical antifungal	NB-UVB 36 (3x/wk)	CR

5 (Figure 5)	29/24	F	PV, vitiligo	Trunk, all 4 limbs	40%	None	1B	Topical antifungal	NB-UVB 32 (3x/wk)	PR

M—male, F—female, MF—mycosis fungoides, NB-UVB—narrowband ultraviolet B, PUVA—psoralen plus ultraviolet A, PV—Pityriasis versicolor, PLC—pityriasis lichenoides chronica, PMH—progressive macular hypomelanosis, NR—no clinical response, PR—partial clinical response, CR—complete clinical response.

## References

[1] Stone ML, Styles AR, Cockerell CJ, Pandya AG (2001). Hypopigmented mycosis fungoides: a report of 7 cases and review of the literature. *Cutis*.

[2] Whitmore SE, Simmons-O’Brien E, Rotter FS (1994). Hypopigmented mycosis fungoides. *Archives of Dermatology*.

[3] Akaraphanth R, Douglass MC, Lim HW (2000). Hypopigmented mycosis fungoides: treatment and a 6 (1/2)-year follow-up of 9 patients. *Journal of the American Academy of Dermatology*.

[4] Sezer E, Sezer T, Senayli A, Koseoglu D, Filiz N (2006). Hypopigmented mycosis fungoides in a Caucasian child. *European Journal of Dermatology*.

[5] Ardigó M, Borroni G, Muscardin L, Kerl H, Cerroni L (2003). Hypopigmented mycosis fungoides in Caucasian patients: a clinicopathologic study of 7 cases. *Journal of the American Academy of Dermatology*.

[6] Qari MS, Li N, Demierre MF (2000). Hypopigmented mycosis fungoides: case reports and literature review. *Journal of Cutaneous Medicine and Surgery*.

[7] Wain EM, Orchard GE, Whittaker SJ, Spittle MF, Russell-Jones R (2003). Outcome in 34 patients with juvenile-onset mycosis fungoides: a clinical, immunophenotypic, and molecular study. *Cancer*.

[8] Tan EST, Tang MBY, Tan SH (2006). Retrospective 5-year review of 131 patients with mycosis fungoides and Sézary syndrome seen at the National Skin Centre, Singapore. *Australasian Journal of Dermatology*.

[9] Zackheim HS, Kashani-Sabet M, Amin S (1998). Topical corticosteroids for mycosis fungoides: experience in 79 patients. *Archives of Dermatology*.

[10] Herrmann JJ, Roenigk HH, Hurria A (1995). Treatment of mycosis fungoides with photochemotherapy (PUVA): long-term follow-up. *Journal of the American Academy of Dermatology*.

[11] Ahmad K, Rogers S, McNicholas PD, Collins P (2007). Narrowband UVB and PUVA in the treatment of mycosis fungoides: a retrospective study. *Acta Dermato-Venereologica*.

[12] Ghodsi SZ, Hallaji Z, Balighi K, Safar F, Chams-Davatchi C (2005). Narrow-band UVB in the treatment of early stage mycosis fungoides: report of 16 patients. *Clinical and Experimental Dermatology*.

[13] Boztepe G, Sahin S, Ayhan M, Erkin G, Kilemen F (2005). Narrowband ultraviolet B phototherapy to clear and maintain clearance in patients with mycosis fungoides. *Journal of the American Academy of Dermatology*.

[14] Hofer A, Cerroni L, Kerl H, Wolf P (1999). Narrowband (311-nm) UV-B therapy for small plaque parapsoriasis and early-stage mycosis fungoides. *Archives of Dermatology*.

[15] Gökdemir G, Barutcuoğlu D, Sakiz D, Köşlüt A (2006). Narrowband UVB phototherapy for early-stage mycosis fungoides: evaluation of clinical and histopathological changes. *Journal of the European Academy of Dermatology and Venereology*.

[16] Reddy K, Bhawan J (2007). Histologic mimickers of mycosis fungoides: a review. *Journal of Cutaneous Pathology*.

[17] El-Darouti MA, Marzouk SA, Azzam O (2006). Vitiligo vs. hypopigmented mycosis fungoides (histopathological and immunohistochemical study, univariate analysis). *European Journal of Dermatology*.

[18] Petit T, Cribier B, Bagot M, Wechsler J (2003). Inflammatory vitiligo-like macules that simulate hypopigmented mycosis fungoides. *European Journal of Dermatology*.

[19] Kumarasinghe SPW, Tan SH, Thng S, Thamboo TP, Liang S, Lee YS (2006). Progressive macular hypomelanosis in Singapore: a clinico-pathological study. *International Journal of Dermatology*.

[20] El Shabrawi-Caelen L, Cerroni L, Medeiros LJ, McCalmont TH (2002). Hypopigmented mycosis fungoides: frequent expression of a CD8+ T-cell phenotype. *American Journal of Surgical Pathology*.

[21] Singh ZN, Tretiakova MS, Shea CR, Petronic-Rosic VM (2006). Decreased CD117 expression in hypopigmented mycosis fungoides correlates with hypomelanosis: lessons learned from vitiligo. *Modern Pathology*.

